# Advancements in vaginal microbiota, *Trichomonas vaginalis*, and vaginal cell interactions: Insights from co-culture assays

**DOI:** 10.15698/mic2025.05.849

**Published:** 2025-05-15

**Authors:** Fernanda Gomes Cardoso, Tiana Tasca

**Affiliations:** 1 Faculdade de Farmácia and Centro de Biotecnologia, Universidade Federal do Rio Grande do Sul, Porto Alegre, RS, Brazil.

**Keywords:** Candida spp., co-culture, Lactobacillus spp., Trichomonas vaginalis, vaginal health, vaginal microbiota

## Abstract

Vaginal microbiota involves seven communities-state types (CST), four dominated by *Lactobacillus*. *L. crispatus*, particularly, offers enhanced protection against infections. Recurrent vulvovaginal candidiasis and trichomoniasis affect millions of people annually, often asymptomatically, facilitating infection spread and leading complications. Co-culture, the technique of cultivating different microbial populations together to mimic real-life conditions, enables the study of microorganism interactions, including inhibitory or promotive effects on pathogens. This review compiles data on co-culture techniques to analyze interactions among *Lactobacillus *spp., *Candida *spp., and *Trichomonas vaginalis*. PubMed was searched using medical subject headings (MESH) terms, ‘co-culture’, ‘coculture,’ ‘cocultivation,’ ‘co-incubation,’ and ‘*Trichomonas vaginalis*’, ‘*Candida *spp.’, ‘*Lactobacillus *spp.’. Articles were selected based on relevance to vaginal health, English language, availability, and use of co-culture or co-incubation techniques in the past 24 years. Co-culture and co-incubation studies over the past 24 years have advanced our understanding of microbiota-host, pathogen-host, and pathogen-host-microbiota interactions. These studies reveal that microbiota composition impacts infections, with the microbiota producing substances against pathogens and pathogens developing stress tolerance mechanisms. They elucidate pathogen virulence factors, interactions with immune cells, and how ecological relationships between microorganisms can enhance pathogenicity.

## Abbreviations

AMPs - antimicrobial peptides,

BV - bacterial vaginosis,

CFU - colony forming unit,

CLM - communal liquid growth medium,

CLSM - confocal laser scanning microscopy,

CSTs - communities-state types,

GBS - Group B Streptococcus,

hECs - ectocervical cells,

ROS - reactive oxygen species,

SEM - scanning electron microscopy,

STI - sexually transmitted infection,

VVC - vulvovaginal candidiasis.

## INTRODUCTION

The human body harbors a complex microbial community, known as microbiota, which plays a crucial role in influencing overall health, including functions related to immunity, nutrition, and disease susceptibility 1. In the vaginal environment, factors such as oxygen levels, glucose, iron, and nutrients create conditions conducive to supporting diverse microbial populations 2. Culture-dependent techniques have long studied vaginal microbiota, but OMICS technologies now allow for the identification of previously uncultivable microorganisms. This advancement has characterized the vaginal microbiota into seven community-state types (CSTs), with four dominated by *Lactobacillus *species. CST-I is dominated by *Lactobacillus crispatus, *CST-II by* Lactobacillus gasseri*, CST-III by *Lactobacillus iners*, and CST-V by *Lactobacillus jensenii* 3. The presence of *L. crispatus* is particularly associated with enhanced protection against infections 4. In contrast, a dysbiotic vaginal microbiota lacking *Lactobacillus *enriched with anaerobes can lead to bacterial vaginosis and is linked to infections like trichomoniasis and vulvovaginal candidiasis (VVC) 5,6.

The vaginal microbiota includes a fungal community with *Candida albicans* as the most prevalent species, which can colonize without causing infection 7. The presence of *C. albicans *increases the risk of vaginitis due to immune imbalance, dysbiosis, or epithelial barrier breaches. This opportunistic pathogen can proliferate, leading to VVC, the second most common cause of vaginitis 8. Recurrent vulvovaginal candidiasis (RVVC), often caused by *C. albicans, *affects 138 million women annually and severely impacts quality of life and incurs high costs 9. VVC has demonstrated an escalation in drug resistance, particularly against the primary therapeutic option, fluconazole 10. This characteristic, where *Candida *can either be a part of women’s normal microbiota or act as the etiological agent of VVC, is attributed to its dimorphic nature 11.

The first line of defense against *C. albicans *is mediated by the innate immune system, which recognizes the fungus through phagocytic receptors 12. Neutrophils and macrophages engulf *C. albicans *by phagocytosis, trapping it within a phagosome and exposing it to antimicrobial peptides (AMPs), reactive oxygen species (ROS), and other fungicidal factors 13,14. However, *C. albicans *has developed mechanisms to evade the immune detection, escape from phagosomes, and resist immune cell killing 15. Additionally, epithelial cells secrete AMPs and pro-inflammatory cytokines, which play a crucial role in recruiting neutrophils to the infection site 16,17. To survive this, *C. albicans *produces candidalysin, a toxin that damages host cell, further complicating the immune response 18.

Trichomoniasis, another form of vaginitis, is the most common non-viral sexually transmitted infection (STI), caused by the flagellated protozoan *Trichomonas vaginalis*, with an estimated annual incidence of 156 million new cases 19. Approximately 80% of individuals infected are asymptomatic or experience minimal symptoms, leading to complications for affected individuals and facilitating the spread of the protozoan 20. These complications stem from the intricate parasite-host relationships, driven by *T. vaginalis *capability to engage in phagocytosis, cytoadherence, and cytotoxicity, whether through contact-dependent or independent-contact mechanisms 21. Concerningly, resistance of *T. vaginalis *isolates to 5-nitroimidazole-class drugs, the current treatment of choice, was documented merely three years after FDA approval for their use 22.

Similar to *C. albicans, *the initial immune response against *T. vaginalis* is mediated by innate immune system, involving neutrophils, myeloid cells, and the complement system 23. *T. vaginalis *employs immune modulation to establish and sustain infection. Its interaction with neutrophils reduces chemokine production and immune cell recruitment, while also promoting neutrophil apoptosis and increasing ROS production 24. To colonize the urogenital tract, *T. vaginalis *needs to adhere to vaginal epithelial cells through degrading the protective mucus layer 25. Following adhesion, the parasite initiates cytotoxicity, a process involving cytolysis, phagocytosis, and the disintegration of the epithelial monolayer, facilitating nutrient acquisition and immune evasion 26.

This review updates co-culture and co-incubation techniques for studying interactions of *Lactobacillus *spp., representing a predominant member of the healthy vaginal microbiota; *Candida *spp., the most abundant yeast in the vagina, and *T. vaginalis*, responsible for the most widespread nonviral STI worldwide. These microorganisms were co-incubated or co-cultured with other microorganisms related to STIs, vaginal microbiota, vaginal cells, or immune cells to assess vaginal health. This review explores two approaches: communal liquid medium growth (CLM), which includes direct mixing or biofilm formation, and membrane separation methods like the Transwell® system. **Table 1** summarizes these studies. We define co-incubation as short-term interactions (up to 24 h) and co-culture as longer interactions (over 24 h), considering the differences in doubling rates between the studied microorganisms and human cells 27,28. *T. vaginalis *replicates every 4-6 h 29, *C. albicans *every 3-4 h 30, and *Lactobacillus *species approximately every 1 h 31, whereas human cells have a slower doubling time of around 24 h 32. The terms co-culture and co-incubation were retained as used by the original authors, even if their application was inconsistent.

**Table 1:** Summary co-incubation/co-culture studies on sexually transmitted infections (STIs) microorganisms, vaginal microbiota, vaginal cells, and immune cells. BV, Bacterial vaginosis; VVC, Vulvovaginal candidiasis; HeLa, Cervical cancer cell; TV, *Trichomonas vaginalis*; SEM, Scanning electron microscopy; TEM, Transmission electron microscopy; CFU, Colony forming units assay; CLSM, Confocal laser scanning microscopy; ECL, Electrochemiluminescence; ELISA, E*nzyme-linked immunosorbent assay*; LDH, Lactate dehydrogenase assay; PCR, Polymerase chain reaction; RT-qPCR, Reverse transcription and quantitative PCR; TvMIF, *T. vaginalis* macrophage migration inhibitory factor; XTT, 2,3-Bis-(2-Methoxy-4-Nitro-5-Sulfophenyl)-2*H*-Tetrazolium-5-Carboxanilide; MTT, 3-(4,5-dimethylthiazol-2-yl)-2,5-diphenyltetrazolium bromide; H_2_O_2, _Hydrogen peroxide; GBS, Group B *Streptococcus*; CFS, Cell-free supernatants; Ref., References.

Microorganisms/Disease	Host Cell type	Co-incubation or Co-culture?	Techniques for evaluating microbial cultures	Interaction	Key findings	Ref.
*Pseudomonas aeruginosa*,* Candida albicans*, *C. tropicalis*, *C. parapsilosis*, *C. krusei*, *C. dubliniensis* and *C. glabrata*/Mixed biofilms	-	Co-culture	CFU counts CLSM SEM	Dual species biofilm (Bacteria-Yeast)	Mutual inhibition of biofilm formation	61
*T. vaginalis*, *Gardenerella vaginalis, Prevotela bivia, Lactobacillus acidophilus, L. crispatus* and *L. jensenii*/Trichomoniasis and BV	Epithelial cells lines of vagina, ectocervix and endocervix	Co-culture	Colorimetric assay ECL assay Quantikine ELISA	Pathogen-Host Cells-Microbiota	Antagonist interaction: TV vs. *Lactobacillus* spp. Synergic interaction: TV vs. bacteria BV-associated	50
*G. vaginalis, Candida albicans, L. plantarum*, and *L. fermentum*/BV and VVC	HeLa	Co-incubation (Pre-treatment post-treatment)	ELISA Luciferase assay	Microbiota-Host cells	Anti-inflammatory effect of *Lactobacillus* spp.	54
*C. glabrata* and *L. fermentum, L. casei, L. crispatus, L. paracasei, L. gasseri*, and *L. Rhamnosus*/VVC	-	Co-culture	Fluorescence microscopy CFU counts	Pathogen-Microbiota	Mechanism by Candida for tolerating environmental stress	41
*T. vaginalis* and *Escherichia coli/Trichomoniasis*	-	Co-incubation	RT-qPCR	Pathogen-Microbiota	Mechanism by TV interacts with microbiota	47
*T. vaginalis*/Trichomoniasis	-	Transwell co-culture	Flow cytometry Gene knockout and adding back of TvMIF in TV	Pathogen-Pathogen	Mechanism of TV survival under nutrient stress	71
*C. albicans, L. gasseri, L. rhamnosus, L. acidophilus*, and *L. paracasei*/VVC	-	Co-incubation Pre-incubation	Antagonism assay Crystal violet assay	Pathogen-Microbiota	Antifungal and antivirulence activity of *Lactobacillus* spp.	40
*C. albicans, G. vaginalis*, and *Chlamydia trachomatis*/Chlamydia infection	HeLa	Transwell co-culture	Crystal violet assay	Microbiota-Pathogen-Host cell	*C. albicans* and *G. vaginalis* biofilms as a reservoir of *C. trachomatis*	64
*Streptococcus agalactiae, L. reuteri, L. gasseri*, and *L. crispatus*/Neonatal infections	Human endometrial stromal cells	Co-culture	CFU counts Fluorescent nucleic acid stain	Microbiota-Pathogen-Host cell	Secreted products of *Lactobacillus* spp. inhibited GBS growth, biofilm formation and invasion of host cells	53
*G. vaginalis* and *L. crispatus*/BV	Epithelial cells lines of vagina, ectocervix and endocervix	Co‐culture	Imaging LDH assay qPCR ELISA	Microbiota-Pathogen-Host cell	G. vaginalis induces cell death and immune responses, but not L. crispatus	55
*C. parapsilosis, L. acidophilus, L. plantarum, L. rhamnosus*, and *L. reuteri*/VVC	Vaginal epithelial squamous cell carcinoma	Transwell co-culture	Imaging LDH assay XTT analysis	Pathogen-Microbiota	CFS of Lactobacillus spp. inhibits *in vitro* infection by *C. parapsilosis*	68
*C. albicans* and *E. coli*/VVC	-	Co-culture	Crystal violet XTT assays	Dual species biofilm (Yeast- Bacteria)	E. coli modulates biofilm of *C. albicans*	58
*T. vaginalis, L. gasseri*, and* G. vaginalis*/Trichomoniasis and BV	-	Co-incubation	CFU counts RT-qPCR Enzymatic assay	Microbiota-Pathogen	Mechanism of TV interaction with microbiota	48
*C. albicans, Rhodotorula mucilaginosa, Malassezia furfur*, and *Naganishia albida*/VVC	-	Co-culture	Crystal violet assay MTT assay Hemocytometer counts	Dual species biofilm	Ecological relationship within yeast microbiota enhances pathogenicity during co-culture	59
*L. crispatus, L. gasseri*, and *L. jensenii* and *G. vaginalis*/BV	-	Transwell Co-culture	TEM Quantification of L- and D-lactic acid and H_2_O_2_	Pathogen-Microbiota	*L. jensenii* produces a bactereriocin-like substance that inhibits *G. vaginalis*	67
*T. vaginalis, G. vaginalis, P. bivia*, and *Atopobium vaginae* /BV and Trichomoniasis	Ects	Transwell co-culture	Fluorescence qPCR LDH assay	Pathogen-Microbiota-Host cell	Mechanism of cytotoxicity by TV and BV-associated bacteria	66
*C. trachomatis, L. crispatus, L. jensenii, L. gasseri, L. iners*, and *G. vaginalis/ C. trachomatis* infection	Fibroblasts and cervical epithelial cells	Transwell system	Lactic acid measure Immunofluorescence staining Imaging Confocal	Pathogen-Microbiota-Host cell	Microbiota modulates *C. trachomatis *infection	65
*T. vaginalis, S. agalactiae*, and *L. iners*/vaginitis	-	Co-culture	CFU counts Hemocytometer counts	Pathogen-Microbiota	Impact of microbiota composition on *T. vaginalis* infection	43
* L. crispatus* and *C. albicans*/CVV	-	Transwell co-culture	CFU counts	Yeast-Microbiota	Mechanism by *C. albicans* alkalinizes an acidic environment	74

PubMed was chosen as the information source, and Medical Subject Headings (MESH) terms were used to search the vocabulary thesaurus. The keywords and search strategy applied were: “Co-culture” OR “Coculture” OR ‘Cocultivation’ OR ‘Co-incubation’ AND ‘*Trichomonas vaginalis’ *OR ‘*Candida *spp.’ OR ‘*Lactobacillus *spp.’. This search yielded 365 articles, of which 19 were selected for this review. The selection criteria required articles to be relevant to vaginal health, written in English, and accessible. The screening process involved examining the materials and methods sections, and only studies employing co-culture or co-incubation techniques within the past 24 years were included.

## CO-CULTURE APPLILCATONS IN THE STUDY OF VAGINAL HEALTH

Co-cultures techniques enable the simultaneous cultivation of different cell populations, offering advantages over monocultures by more accurately mimicking real-life conditions 33. This approach allows for the study of both cell-cell and drug-cell interactions, the latter being particularly valuable for drug research by providing an *in vivo*-like cell culture model 34. Additionally, co-culture methods facilitate the investigation of microbial interactions, helping to elucidate patterns of interaction and the ability of resident microbiota to either inhibit or promote the growth of pathogens or opportunist organisms 35. Such studies are particularly relevant in microbiota - host - pathogen interactions. The most used techniques include CLM and membrane separation, particularly Transwell system.

### Communal liquid medium growth (CLM)

CLM is widely used to study microbial interactions, substance production, and over- or under-yielding in co-culture by varying inoculation ratios and incubation times 36. In this system, direct mixing enhances interactions and molecular exchange compared to monoculture 37. Alternatively, biofilm formation with multiple species in the same medium allows for the exploration of regulatory factors and microorganism interrelationships 38.

Among the key microbial players in the vaginal environment, *Lactobacillus *spp. play a crucial role in maintaining an acidic pH producing lactic acid, which restricts the growth of opportunistic microbes. Beyond acidification, lactobacilli inhibit pathogen adherence, limit nutrient availability, and exert a fungistatic effect on *Candida *spp., preventing yeast overgrowth 39.

The protective role of *Lactobacillus *spp. is further supported by studies on their antimicrobial metabolites. Itapary dos Santos *et al. *(2019) demonstrated the antifungal and antivirulence potential of metabolites produced by vaginal lactobacilli, including *L. *gasseri, *Lactobacillus rhamnosus, Lactobacillus acidophilus *and *Lactobacillus paracasei*. Among the 20 strains tested, 15 secreted biosurfactants that reduced *C. albicans *biofilm formation and adhesion 40. In contrast, another study identified a mechanism that enables *C. glabrata—*the second most common species associated with VVC—to withstand environmental stressors such as osmotic stress, low pH, and exposure to carboxylic acids. The virulence of *C. glabrata *is linked to the high osmolarity glycerol (HOG) response pathway, which enhances its survival under acidic conditions created by lactic and other weak carboxylic acids from *Lactobacillus *spp., as well as provides protection against oxidative stress induced by macrophage activity 41*. *

However, not all *Lactobacillus *species contribute equally to vaginal health. *L. iners*, for instance, lacks the capacity to produce hydrogen peroxide, and its lactic acid production is significantly diminished 42. A study demonstrated that in a co-culture with *T. vaginalis*, *L. iners *initially exhibited reduced growth. However, it appears to adapt to the stress conditions imposed by the protozoan and can survive prolonged incubation periods 43. *L. iners *is frequently found in the vaginal microbiota of women with bacterial vaginosis 44 and, due to its weak probiotic activity, a microbiota dominated by *L. iners *is associated with STIs and adverse pregnancy outcomes 45.

Bacterial vaginosis (BV) affects reproductive-age women, with 50% of cases being asymptomatic, increasing the risk of acquiring HIV, *Neisseria gonorrhea*, *T. vaginalis*, and other STIs 46. A study using a bacterial colonization model explored the interaction between BV-associated bacteria and *T. vaginalis*. Epithelial cells were colonized by *Lactobacillus *spp., *Gardnerella vaginalis, *or *Prevotella bivia*, then co-infected with *T. vaginalis*. The results showed that *T. vaginalis *reduced colonization of epithelial cells by *L. acidophilus*, *Lactobacillus crispatus*, and *L. jensenii*. Additionally, the interaction between *G. vaginalis*, *P. bivia*, and *T. vaginalis *was found to modulate the immune system, amplifying pro-inflammatory responses 47.

Beyond interactions with lactobacilli, *T. vaginalis *also engages in cooperative relationships with anaerobic bacteria such as *G. vaginalis, *Atopobium vaginae**, and *P. bivia 48. *This cooperation is likely driven by lateral gene transfer, through which *T. vaginalis *acquired genes from the N1pC/P60 family, known for their role in bacterial peptidoglycan hydrolysis. Co-culture studies with *Escherichia coli*, a vaginal microbiota member, have shown that *T. vaginalis *exploits bacterial interactions to enhance infection progression. In the presence of bacteria, N1pC/P60 proteins in *T. vaginalis* are upregulated, increasing protozoan persistence in mixed cultures 49.

Additionally, co-incubation of *T. vaginalis *with *L. gasseri* reduces bacterial colony-forming units (CFU), triggered protozoan aggregation, and induced upregulation of TvN1pC gene expression. However, these proteins did not inhibit the growth of* G. vaginalis*, suggesting a specific role in disrupting vaginal microbiota balance 50. This study establishes N1pC/P60-containing peptidases as key effectors in *T. vaginalis* interactions with the vaginal microbiota, particularly lactobacilli. These enzymes facilitate bacterial degradation both extracellularly and post-phagocytosis, reinforcing the parasite’s ability to manipulate its microbial environment for survival and persistence.

Neonatal infections caused by Group B *Streptococcus *(GBS) are linked to an increased risk of premature birth and stillbirth 51. GBS can asymptomatically colonize pregnant women, creating a risk of transmission to newborn during childbirth, particularly by aspiration 52. A study investigating the influence of the vaginal microbiota on invasive GBS strains their interaction with human endometrial cells in co-culture with various *Lactobacillus *strains and their supernatants. While live *Lactobacillus *spp. did not significantly impact GBS growth or biofilm formation, the secreted supernatants effectively inhibited GBS growth, biofilm development, and cellular invasion 53.

The vaginal microbiota plays a crucial role in protecting against pathogens, though its effectiveness depends on the specific microorganisms present. A cell model infected with *G. vaginalis *or *C. albicans *was used to evaluate the immunomodulatory effects of two *Lactobacillus *species. The results demonstrated that *Lactobacillus *spp. reduced the release of pro-inflammatory cytokines and suppressed NF-kB activation, a process typically triggered when *G. vaginalis *or *C. albicans *interact with HeLa cells 54. Similarly, a co-culture model of *L. crispatus *or *G. vaginalis* with endocervical, ectocervical or vaginal cells revealed that *G. vaginalis *induced cell death, compromised cell integrity, and triggered an epithelial immune response through NF-kB activation and increased pro-inflammatory cytokine release. In contrast, *L. crispatus *maintained epithelial barrier integrity and did not induce inflammatory response 55.

Microbial interactions within the vaginal microbiota further influence infection dynamics, particularly through biofilm formation, a process that enhances survival and resistance to antimicrobial agents. Many fungi, including those from the genera *Candida, Clavispora, Malassezia, Rhodotorula, Aspergillus, *and *Leptosphaerulina*, coexist in this environment, fostering interactions that may contribute to pathogenicity 56,57. *Candida *species, for instance can form heterogeneous biofilms with other microorganisms 58. *C. albicans *has been shown to interact with *Malassezia *spp*., *suggesting a symbiotic relationship characterized by increased fungal growth, mixed biofilms, and enhanced germ tube formation during co-culture 59.

Understanding microbial interactions within biofilms is essential, as biofilms exhibit inherent resistance to antimicrobial agents, complicating the treatment of co-occurring infections 60. In a dual-species biofilm involving various *Candida *species and *Pseudomonas aeruginosa*, the presence of bacteria inhibited biofilm formation, as indicated by a reduction in CFU for both *Candida *spp. and *P. aeruginosa*. The sparse biofilm architecture was confirmed using scanning electron microscopy (SEM) and confocal laser scanning microscopy (CLSM) 61. Another study assessed the effect of *E. coli *on *C. albicans *biofilm formation using vaginal swab isolates. The results showed a reduction in biofilm formation, as observed through CLSM and SEM, suggesting that *E. coli *may function as a microbial reservoir that influences *C. albicans *biofilm development 62*. *

### Permeable membrane technique

The permeable membrane technique (Transwell system®) utilizes a permeable membrane in multi-well plates to separate cells, enabling the exchange of signaling molecules without direct contact 38. This polycarbonate membrane divides the system into an upper insert and a lower reservoir. While the system is easy to set up and requires only a small culture volume, careful selection of membrane porosity is essential to ensure proper metabolite diffusion. This co-culture method mimics *in vivo *conditions, facilitating the identification of extracellular molecules released during interactions 63*. *Leveraging this system allows for deeper insights into host-pathogen interactions, the antimicrobial properties of *Lactobacillus *spp., and the persistence mechanisms of vaginal pathogens.

The interaction between *Chlamydia trachomatis* and biofilms of *C. albicans *or *G. vaginalis* was investigated using a Transwell co-culture system, where biofilms were placed on the upper platform and a Hela cell monolayer on the lower part. The results demonstrated that *C. trachomatis *could survive within the biofilm while still inducing inclusions in the cell monolayer, confirming that it retains its infectious proprieties within the biofilm 64. Further studies using a three-dimensional cervical epithelium model, composed of fibroblasts, epithelial cells, and various *Lactobacillus *species, revealed that *Lactobacillus *spp. producing D-lactic acid provided protection against *C. trachomatis*. In contrast, *L. iners*, which does not produce this isoform, was associated with increased susceptibility to infection, suggesting that women with an *L. iners*-dominated microbiota are more vulnerable to chlamydial infection 65.

A polymicrobial infection model employing the Transwell system assessed the influence of *T. vaginalis *and BV-associated bacteria on the paracellular permeability of ectocervical cells (hECs). The BV-associated bacteria including *G. vaginalis*, *P. bivia, *and *A. vaginae*, are key members of the CST-IV vaginal microbiota group. The study found that these microorganisms collectively increased the paracellular permeability of hECs, elevated phosphatase activity - indicating compromised monolayer integrity - and upregulated the expression of tight junction proteins and pro-inflammatory cytokines (IL-6 and TNF- α). These findings underscore the microbiota’s significant role in trichomoniasis pathogenesis 66.

The antimicrobial potential of *Lactobacillus *spp. against vaginal pathogens has also been explored using Transwell co-culture systems. One study found that *L. jensenii *produces a bacteriocin-like substance with specific bactericidal activity against *G. vaginalis, Gardnerella piotii, *and *Gardnerella leopoldii*, without affecting the growth of other uropathogens or *Lactobacillus *species such as *L. crispatus *and *L. gasseri *67*.* Another study evaluated the antimicrobial properties of *L. acidophilus, Lactobacillus plantarum, L. rhamnosus*, and *Lactobacillus reuteri* against *Candida parapsilosis* in a co-culture system comprising vaginal epithelial cells, bacteria, and yeast. The results demonstrated that all tested *Lactobacillus *species inhibited the virulence factors of *C. parapsilosis *by reducing its proliferation, viability, and metabolic activity, suggesting a postbiotic-like protective effect against those mucosal infections 68.

The pathogens have acquired mechanisms for persistence in the vaginal environment though interactions with the host. Human macrophage migration inhibitory factor (huMIF) plays a crucial role in regulating cell growth and survival 69. *T. vaginalis *shares a homologous protein with its host, known as TvMIF, which enhances the protozoan’s survival during nutrient starvation 70,71.

To investigate the role of this protein, a Transwell co-culture system was employed, in which parasites overexpressing TvMIF were separated by a membrane from those carrying an empty vector. Under serum starvation, the TvMIF-overexpressing parasites exhibited significantly increased survival, whereas TvMIF knockout reduced parasite viability. This survival mechanism is activated under nutrient deprivation, as TvMIF expression and secretion are upregulated. Secreted TvMIF enhances parasite survival through an intracellular positive-feedback loop that promotes its own expression and secretion. Additionally, TvMIF inhibits apoptosis, facilitating parasite persistence in the urogenital tract — an otherwise unfavorable environment. Chen *et al*. (2018) demonstrated that this conserved protein plays a crucial role in parasite survival and mediated host-pathogen crosstalk 71. The metabolic activities of vaginal microorganisms further shape microbial interactions. *C. albicans*, for example, metabolizes amino acids, leading to ammonia accumulation and subsequent alkalinization of the vaginal environment 72. This pH shift promotes *C. albicans*’ transition from yeast to filamentous growth, a key virulence factor 73. A study using a Transwell co-culture system examined whether ammonia production by *C. albicans *affects the growth of *L. crispatus, *a known fungal antagonist. The results showed that *L. crispatus *growth remained unaffected by the alkalinization caused by *C. albicans*, suggesting that other mechanisms contribute to dysbiosis in the vaginal ecosystem 74.

By utilizing Transwell-based co-culture models, researchers continue to uncover the complex interplay between the vaginal microbiota and pathogens, shedding light on protective and pathogenic mechanisms that influence vaginal health.

## CONCLUSION

Co-culture systems provide valuable insights into microbial interactions, including gene and protein crosstalk, the influence of microbiota on pathogen persistence within the host, producing antimicrobial substances or disease-promoting factors. Over the past 24 years, co-culture and co-incubation techniques have significantly advanced our understanding of the complex interactions among microbiota, host cells, pathogens, and biofilms. This review focuses on studies related to trichomoniasis, bacterial vaginosis, chlamydia, neonatal infections, and mixed biofilms, emphasizing the critical role of microbiota composition in infection dynamics and host defense mechanisms. While some microorganisms counteract pathogen colonization, others interact with opportunistic microbes, increasing disease pathogenicity and complicating treatment strategies. Co-culture techniques have been instrumental in studying pathogen virulence factors, immune cells responses to infections, and the ecological interactions that contribute to pathogenicity in the reproductive tract. Furthermore, these platforms could serve as effective models for mimicking the *in vivo* environment in laboratory settings. Moving forward, co-culture models will continue to be a powerful tool for unraveling pathogen dynamics and developing more effective therapeutic approaches.

## CONFLICT OF INTEREST

The authors declare no conflicts of interest.
